# Shape-altering flexible plasmonics of in-situ deformable nanorings

**DOI:** 10.1186/s40580-023-00358-6

**Published:** 2023-03-30

**Authors:** Wei Tao, Florian Laible, Abdelhamid Hmima, Thomas Maurer, Monika Fleischer

**Affiliations:** 1 Institute for Applied Physics and Center LISA+, Eberhard Karls University Tübingen, 72076 Tübingen, Germany; 2 Laboratory Light, Nanomaterials and Nanotechnologies—L2n, University of Technology of Troyes and CNRS EMR 7004, 12 rue Marie Curie, CS 42060, CEDEX, 10004 Troyes, France

**Keywords:** Nanorings, In-situ shape-altering, Electron beam lithography, Wet-etching transfer, Flexible plasmonics

## Abstract

Nanorings (NRs) with their intrinsic cavities have attracted interest as plasmonic nanoparticles for years, due to the uniform electric field enhancement inside the cavity, lower plasmon damping effects and comparatively high refractive index sensitivities. In the present work, we successfully fabricated a series of Au NR arrays on flexible polydimethylsiloxane substrates by taking advantage of state-of-the-art fabrication methods such as electron beam lithography and wet-etching transfer techniques. In-situ optical measurements on these flexible systems are enabled by implementing a homemade micro-stretcher inside an optical reflection spectroscopy setup. The corresponding dark-field spectra of thin-walled NR arrays exhibit a strong shift to longer wavelengths (i.e., ~ 2.85 nm per 1% strain) under polarization perpendicular to the traction, mainly resulting from the increasing shape deformation of the NRs under strain. Moreover, numerical simulations illustrate that the shifting plasmonic mode has a radially-symmetric charge distribution of the bonding mode and is rather sensitive to the tuning of the NRs’ shape as confirmed by a subsequent in-situ scanning electron microscope characterization. These results explore the possibilities of shape-altering flexible plasmonics for nanoparticles with a cavity and indicate potential applications for plasmonic colors and biochemical sensing in future work.

**Graphical Abstract:**

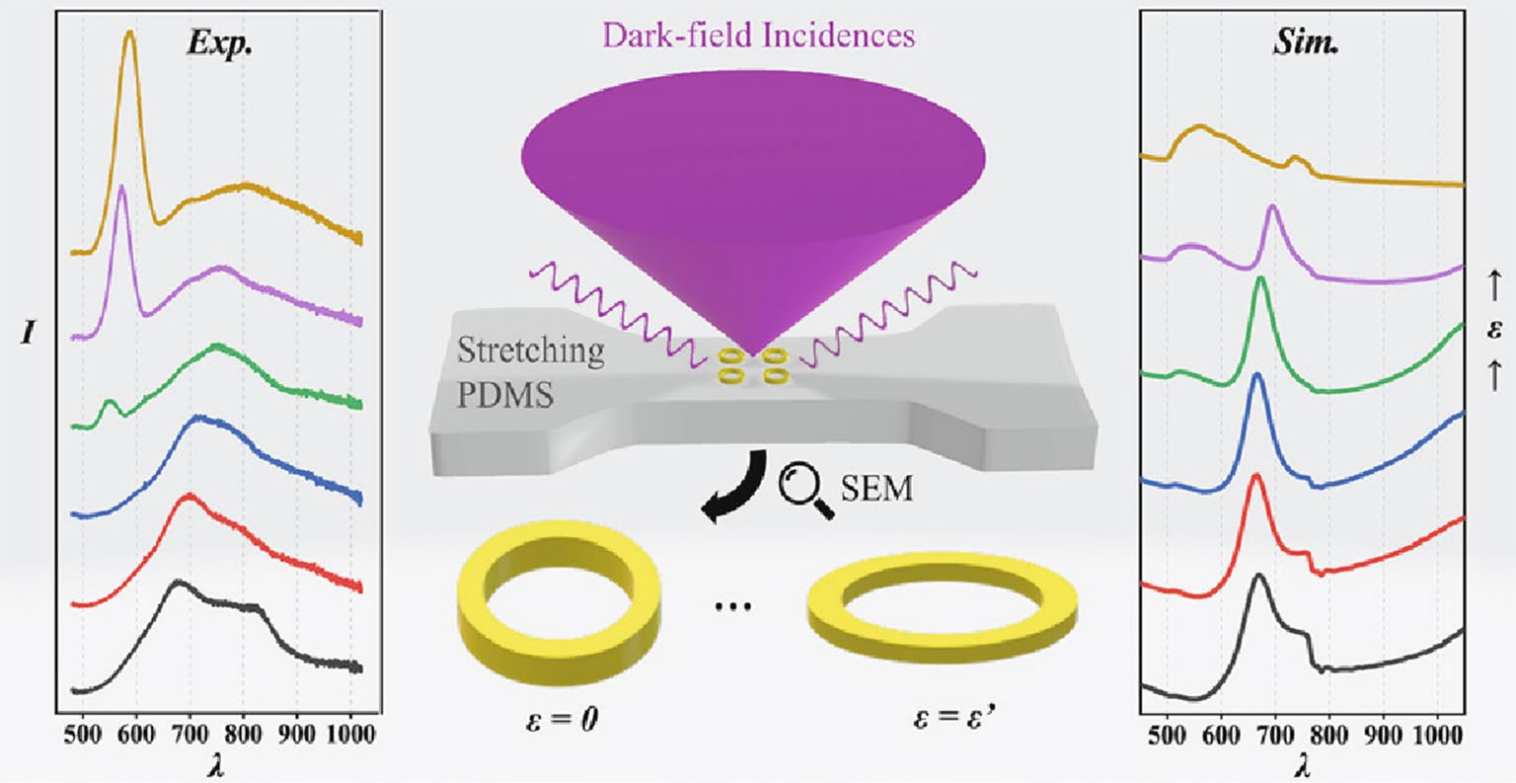

**Supplementary Information:**

The online version contains supplementary material available at 10.1186/s40580-023-00358-6.

## Introduction

Localized surface plasmon resonances (LSPRs), which refer to the light-induced collective oscillation of free electrons at the surface of sub-wavelength nanoparticles (NPs), have shown various appealing applications, such as sensors [1, 2], catalysis [3], photonic elements [4], and molecular electronics [5]. Electromagnetic field confinement effects enable these oscillators to present strong (but also strongly damped) coupling interactions in both near- and far-field regions, depending on the environment’s refractive index, the NP species, geometric parameters, and particularly their shapes [6–8]. In comparison to some ‘solid’ NPs (e.g., discs, triangles, cones, etc.), nanorings (NRs) with their high degree of symmetry and hollow 2D structure have gained increasing interest as the fabrication technology developed from the traditional top-down lithography to recent colloidal synthesis [9–13]. Indeed, ever since pioneers introduced the plasmon hybridization (PH) model to describe the splitting LSPR of the NR modes between the high-energy antibonding and low-energy bonding states [14] plenty of efforts have been made to understand the effect of diameter, thickness, and inter-spacing on their LSPR properties [15, 16]. In general, NRs with different geometries display complex plasmonic modes from the visible to the mid-infrared spectrum, ranging from classical dipolar to multi-polar and cavity modes [17–19].

This inherent easy tuning over a wide wavelength range and the potential for sensing has also inspired researchers to investigate symmetry-reducing effects on the LSPR properties of NRs as the aspect ratios (i.e., ellipticity) increased [20–23]. By using electron beam lithography (EBL) to fabricate a series of square arrays of elliptical NRs (ENRs) on indium-tin-oxide/glass substrates, Tsai et al. reported a strong bonding mode of NRs in the near-infrared region, which redshifted significantly as the aspect ratio increased under both transverse and longitudinal polarizations [24]. Additionally, Cai et al. achieved a controlled tuning of the NR shapes by transferring hexagonal arrays from stretchable polydimethylsiloxane (PDMS) to glass [25]. In their work, similar spectral redshifts of antibonding and multi-polar bonding modes are also observed under both polarizations. However, an obvious drawback for static NRs on rigid substrates is that a systematic variation of the aspect ratio of different NRs will coincide with slight fabrication-related modifications of their geometries, and one can hardly derive the pure effect of the aspect ratio on the LSPR properties. The scientific challenge remains therefore to achieve the in-situ tuning of NRs by overcoming the obstacles that arise in fabrication.

One applicable way is to embed NRs into a flexible substrate. The so-called flexible plasmonics have gained significant progress in the fields of flexible electronics and photonics over the last two decades [26–28]. The main mechanism in such adaptive structures is to monitor the variation of either near-field or far-field coupling effects based on the dynamic tuning of gaps or the stacking of plasmonic NPs under external stimuli applied to their substrate [29, 30]. Therefore, the in-situ tuning of NRs also is promising to pave a new path to shape-altering flexible plasmonics. In this context, its unique elastic and optically transparent properties render PDMS an ideal flexible substrate, but its poor conductivity and hardening effect under electron exposure also hinders direct lithography on the surface [31]. Besides, with an alternative top-down fabrication method by evaporating metals onto PDMS through a carefully designed stencil mask (i.e., nanostencil lithography) it is also difficult to achieve complex cavity structures, such as NRs [32]. More recently, considerable attention has been paid to the pattern transfer techniques on different substrates by designing a series of delicate processes. Du et al. reported a peeling-off transfer approach of lithography-fabricated metallic gratings from silicon to PDMS [33]. Tseng et al. presented a similar work with the aim to transfer aluminum nanostructures [34]. This method also enables the pattern transfer on a wavy surface of PDMS by exploiting the elasticity of PDMS during the modeling process [35, 36].

Here, we adopt an indirect approach to transfer well-ordered Au NR arrays onto PDMS by liquid modeling, in the extension of a previously demonstrated process of a cured transfer where we placed cured PDMS templates on an EBL sample for the modeling and then removed the rigid substrate by etching of a sacrificial layer. In the case of the cured transfer, the NPs rest on top of the flexible PDMS substrate [37]. By in contrast using liquid modeling, a distribution of EBL fabricated NRs is embedded at the PDMS surface with the aim to improve their deformability under strain. Afterward, we measure the optical reflection of those arrays under dark-field illumination with external strain applied in-situ to the substrate. The spectra exhibit apparent merging and redshifting of the LSPR modes in some arrays, and the following in-situ scanning electron microscope (SEM) characterizations and finite-difference time-domain (FDTD) simulations confirm that this is due to shape tuning of the NRs.

## Methods/experimental

### Fabrication

The fabrication process consists mainly of two parts, namely the EBL procedure and the transfer technique. For the EBL, a 200 nm thick sacrificial layer of chromium (Cr) is firstly evaporated onto a cleaned silicon (Si) wafer, resulting in the Cr/Si substrate. Then a 5 v% diluted poly-methyl methacrylate / methyl isobutyl ketone (PMMA/MIBK) resist solution is spin-coated onto the Cr/Si substrate, and a ~ 200 nm PMMA layer is formed after baking at 150 °C overnight. The lithography on the PMMA/Cr/Si substrate is performed with a *JEOL JSM-6500F SEM* together with a *XENOS* pattern generator. Note that one can flexibly tailor the geometry parameters of 2D NPs by adjusting the exposure dose and dwell time through a well-designed pattern file, which is also the prime advantage of EBL. The exposure region for each NR array is (25 μm)^2^, supporting ~ 3600 individual NRs depending on their size and periods. After the exposure, the photoresist is developed via a 25 v% MIBK / isopropyl-alcohol solution, in which the nanoscale exposed areas are thus removed. Then a ~ 50 nm thick Au layer is thermally evaporated onto the above substrate. The following acetone immersion will lift off all the unexposed areas to form the desired Au patterns on the Cr/Si substrate (i.e., EBL sample), due to the strong adhesion between Au and Cr. More details about the EBL procedure can be found in our previous publications [12, 37].

The following transfer procedure includes surface functionalization, PDMS modeling, and wet etching. For the surface functionalization, the linker molecule of (3-Mercaptopropyl) triethoxysilane (MPTS) is introduced to form a stable chemical bond between PDMS and the Au structures [38, 39]. This surface functionalization starts with the immersion of the EBL sample into 5 v% MPTS/n-hexane solution for 2 h [40]. The thiol head group (-SH) from MPTS is prone to binding to the evaporated Au, forming the strong linking group of Au-MPTS. The above sample is then immersed into 50 v% Chloroform/n-hexane solution for at least 3 min [41]. The S_N_2-reaction (i.e., replacing the ethoxy groups of MPTS with hydroxyl groups) takes place in the Chloroform base, which is necessary to bind the PDMS to the Au-MPTS group through a condensation reaction. A few drops of purified water can accelerate the S_N_2-reation. After surface functionalization, the liquid-PDMS modeling is presented as follows. The mixture of PDMS/curing agent (10:1 w/w) is fully degassed and poured onto an extra glass substrate, against which the MPTS/Au/Cr/Si sample is then carefully placed. This system is further degassed, cured at 150 °C for 12 min, and then immersed in the highly selective Cr etchant (*TechniStrip Cr01 solution*) to remove the sacrificial layer and complete the transfer. The etching time varies and depends on the actual conditions, and one can see by eye whether the Cr layer is fully dissolved. Afterward, the Au/PDMS systems are carefully peeled off from their initial Si substrates, rinsed with DI water, dried with N_2_, and finally cut into tensile templates.

### Dark-field reflection measurements

The setup of a *Zeiss Axio Scope A1* is used to measure the reflection spectra of the fabricated samples. The dark field illumination contains a 100 W halogen lamp and a *Zeiss EC EPIPLAN 20x/0.4* objective. The dark field illumination angle is 56.75 ± 2.25°. The back-scattered light is analyzed by a grating spectrometer *LOT SR-303i-B*. The effectively detected spectra range from ~ 480 to 1020 nm. The detection area is ~ (5 μm)^2^, in which many NRs are located such that the grating effect of the NR arrays also needs to be considered. All the measured raw data (*I*_*raw*_) are normalized by the lamp (*I*_*lamp*_), dark current (*I*_*dc*_) and background PDMS (*I*_*bg*_) spectra as follows, such that the observed signal is mainly reduced to the spectra contributed by the NR arrays.

$$I_{nor} = \frac{{I_{raw} - I_{bg} }}{{I_{Iamp} - I_{{{\text{dc}}}} }}$$

### Strain definition

The uniaxial traction is performed via a homemade micro-stretcher placed inside the *Zeiss* setup by means of micrometer screws. As the PDMS is stretched under certain strain values (i.e., external engineering strain), it is observed that the development of the transferred gratings at its surface deviates from the external strain values and rather depends on the local strain, which in turn depends on the geometry and transfer parameters. In order to avoid ambiguity between engineering and local strain applied to the grating, the strain values in this work are defined by the elongation of the outline of the exposed and transferred arrays along the tensile direction measured by the CCD camera in the microscope setup [37].

### In-situ SEM characterization

The SEM characterizations on PDMS are carried out after optical measurement. To reduce charging effects on PDMS, ~ 50 nm Au is sputtered on its surface by an *Agar Sputter Coater* before imaging. Top-view SEM images are taken with a *Philips XL30 FEG*. The same micro-stretcher is now placed inside the SEM chamber. The accelerating voltage is 5.0 kV for PDMS samples, and the fast-mode (screenshot) is applied to avoid artefacts from image drifts in the NRs’ shape variations.

### Numerical simulations

The FDTD simulations are carried out by the commercial software of *Lumerical FDTD solutions*. The geometric dimensions follow the values from the top-view SEM characterizations. The scattering cross-sections are calculated by introducing a total-field scattered-field source, and 3D perfectly matched layer boundary conditions. Gradient meshes are used, with a minimum fine size of 2*2*2 nm^3^. To simplify the annular incidence of the dark-field illumination from the experiment, four separate oblique sources placed at 90° to each other are introduced. θ = 56.75° sets the angle with respect to the injection axis. The refractive index values of Au are taken from Johnson and Christy [42], and we consider a constant refractive index of 1.4 for PDMS based on experience [37]. The surface charge density is analyzed by the pre-set analysis script of the *divergence current* in *Lumerical*.

## Results and discussion

**Fig. 1:**
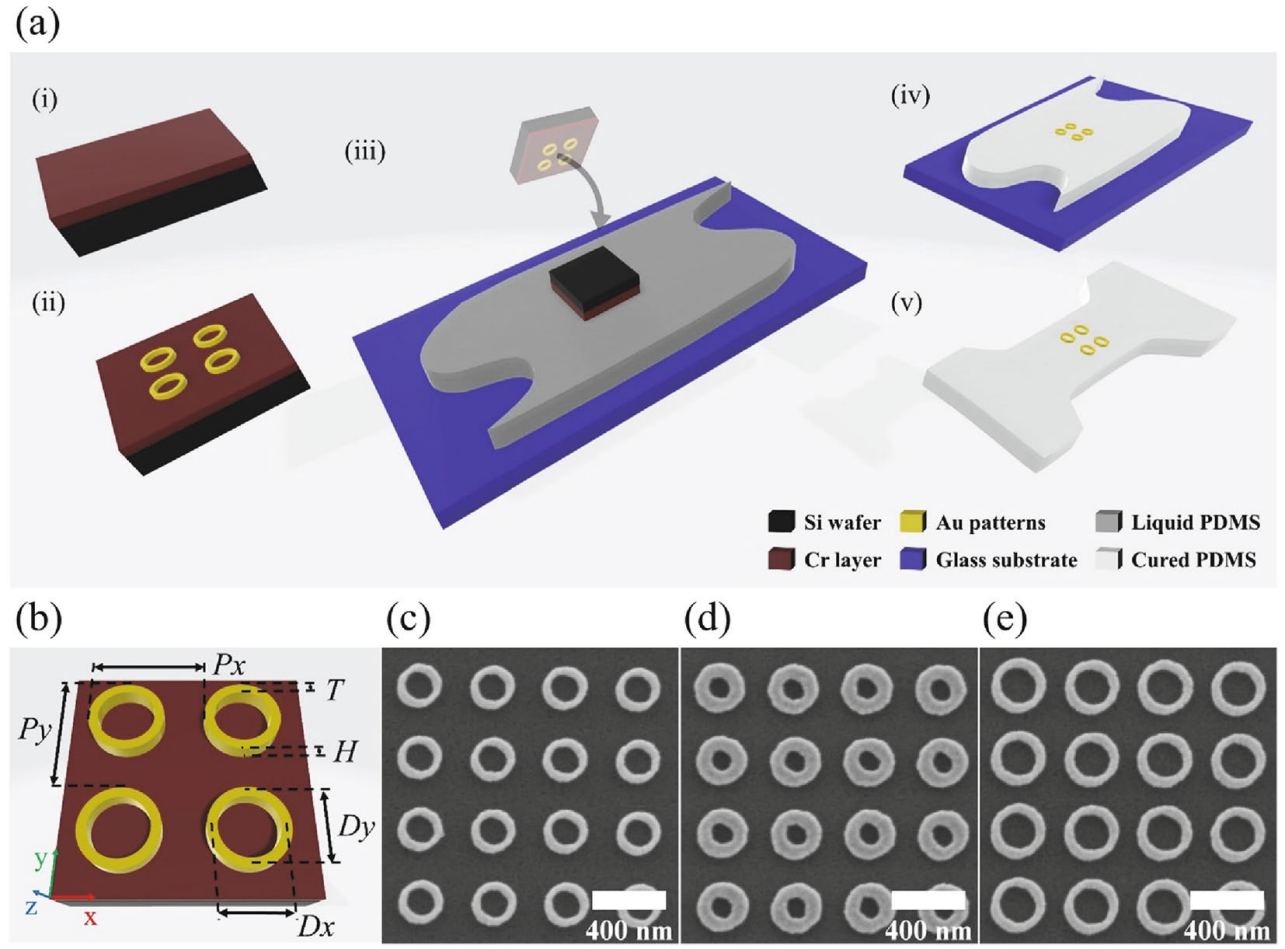
**a** Flow chart of the liquid transfer method. For details, see Methods. **b** Schematic of NR array on Cr/Si substrate with periods Px, Py; average ring diameters Dx, Dy; ring widths (thicknesses) T, and height H. **c**–**e** Top-view SEM images of the three arrays, **c** NR1 with Dx = Dy = 200 nm and T = 49 nm, **d** NR2 with Dx = Dy = 200 nm and T = 83 nm, and **e** NR3 with Dx = Dy = 250 nm and T = 56 nm.

Figure 1a depicts an overview of the pattern transfer technique from the initial EBL template to the final PDMS substrate. The steps from (i) to (ii) represent a classical EBL process on the Cr/Si substrate; step (iii) shows the mechanism of liquid transfer; steps (iii) to (iv) refer to the Cr etching process, and in step (v) the Au/PDMS systems are cut into the desired shape. Figure 1b schematically illustrates the sample layout of an Au NR array deposited on the Cr/Si substrate after the EBL procedure. The geometry of the NR arrays is determined by six parameters, namely the horizontal and perpendicular periods of Px and Py, center diameters of Dx and Dy (measured at the center of the ring width), thickness of T, and height of H. The aspect ratio is defined by the ratio of the NR’s outer diameter in the direction of strain to that perpendicular to the strain, once the NR is transferred onto the stretching PDMS. Since PDMS is usually hardened and also charges under a direct SEM characterization, the geometry parameters are only characterized on the initial Cr/Si substrate before the transfer. Figures 1c, d and e show top-view SEM images of three NR arrays, NR1, NR2 and NR3. Uniform square NR lattices are studied in this work, in which H, Px and Py are fixed at 50 nm, 400 nm, and 400 nm, respectively. In particular, NR1 and NR2 have the same designed center diameter of Dx = Dy = 200 nm, and NR3 displays an increased value of Dx = Dy = 250 nm. Their thicknesses (i.e. ring widths) are namely T_NR1_ = T_avg_ ± SD = 49 ± 7 nm, T_NR2_ = 83 ± 6 nm, and T_NR3_ = 56 ± 6 nm, where T_avg_ and SD are the average and standard deviation values.

**Fig. 2:**
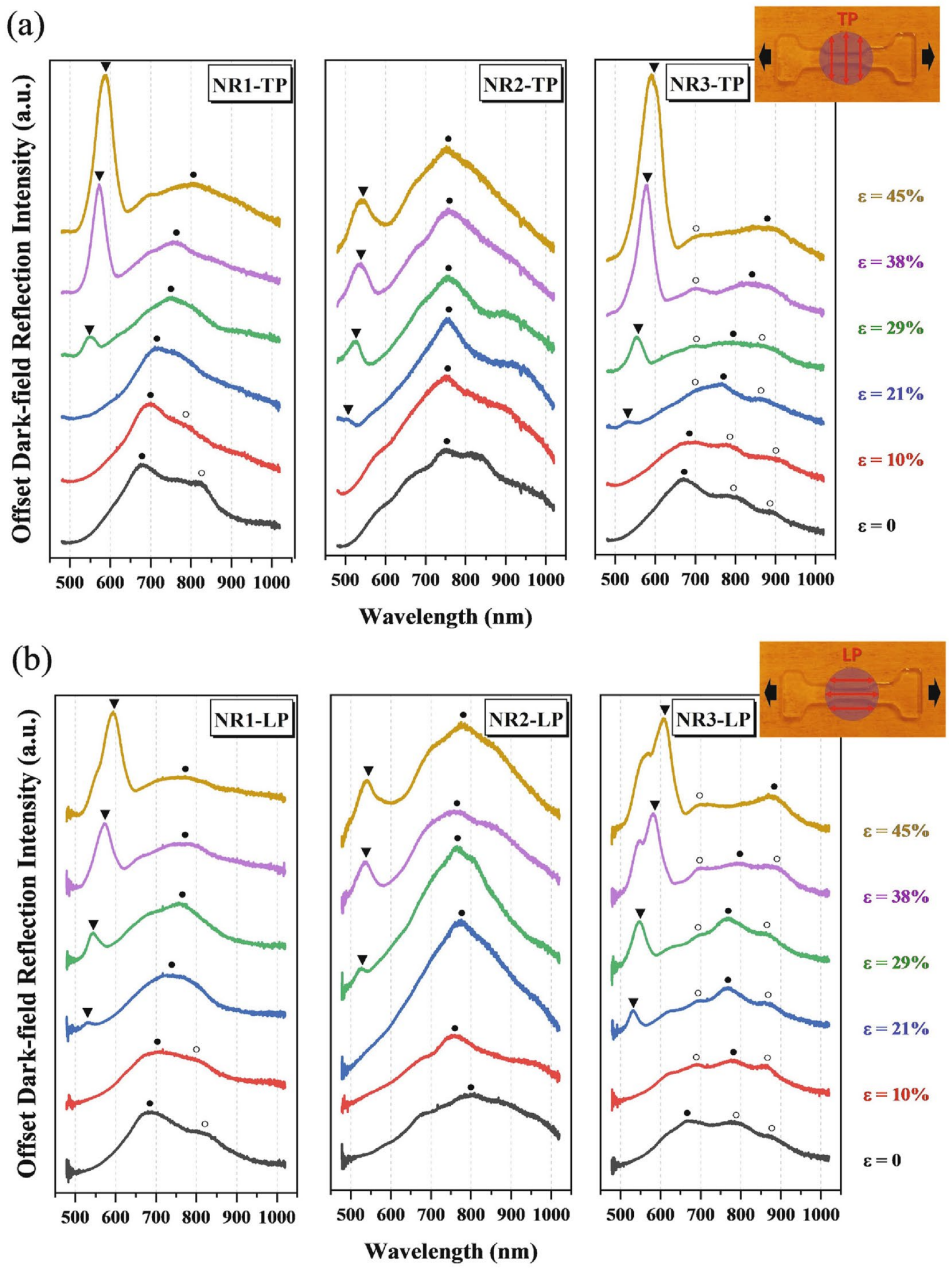
Dark-field reflection spectra for NR1, NR2 and NR3 arrays under both transverse (**a**) and longitudinal polarizations (**b**). The grating and respective highest intensity LSPR modes are marked with triangles and full circles, respectively, where the open circles for the NR1 and NR3 array represent additional LSPR modes that in NR1 are merging. The top-right figures schematically show the polarization conditions and strain applied to PDMS, where the black and red arrows are the stretching and polarization directions, respectively, and the purple circles represent the areas of the top-view dark-field illumination

After fabrication, in-situ reflection spectra are measured under polarized dark-field illumination with a homemade micro-stretcher applied to the PDMS substrate, see Methods [37]. We consider both transverse and longitudinal polarizations (TP and LP), according to whether the polarization of the incident light is oriented perpendicular or parallel to the stretching direction. Figure 2 shows such spectra under different strain values, exhibiting a series of narrow and broad peaks marked with triangles and circles, respectively. Those narrow peaks located at wavelengths from 530 to 600 nm under higher strain (ε > 21%) can be interpreted as the Bragg grating mode [43, 44] where the desired periodicity in combination with the dielectric surrounding satisfies the resonance condition as the strain develops. In Additional file 1: Fig. S1, we perform a second study to distinguish the Bragg and LSPR mode by characterizing the dark-field reflection spectra for three Au disc arrays (with increasing sizes) deposited on an indium tin oxide/glass rigid substrate. As for the Bragg mode shown in Fig. 2, NR1 and NR3 arrays show a similar tuning under strain, whereas the thicker NRs of the NR2 array limit the grating development under strain, leading to a reduced redshift. Additionally, one can also observe a coupling between the Bragg and LSPR modes for NR3-LP under a strain of ε ≥ 38%, resulting in a red-shifting peak splitting off as the strain further develops. This coupling behavior is described as surface lattice resonances [44–46] which is beyond the scope of the current work and should be investigated further.

On the other hand, the spectra in Fig. 2 also present multiple broad LSPR peaks under both polarizations resulting from the dark-field measured under oblique incidence as discussed later [47]. Here, we mark the main LSPR modes of the highest intensity by solid circles. Apart from the main LSPR mode peaking at 680 nm, the NR1 array also shows a separate mode (marked with an open circle) at the strain-less state (ε = 0) under both polarizations, located at 820 nm. These two modes gradually merge into one peak with applied strains, which also redshifts as the strain further increases. Concerning the redshift of the main LSPR modes, one can measure the average shift value for NR1 under transverse polarization (NR1-TP) as 2.85 nm per 1% strain (λ’ = (804–675.8) nm/45% = 2.85 nm/%). Note that this redshift value is higher than the ones observed in traditional gap-altering flexible plasmonic devices, which present up to ~ 2 nm per 1% elongation [29]. By contrast, NR1-LP shows a similar merging and redshift at the beginning, but presents a lower sensitivity to high strain values (ε ≥ 29%). However, the strain-induced spectral behavior significantly varies with NR geometries. To be specific, as the NR thickness increases from ~ 49 nm (NR1) to 83 nm (NR2), the main peak (λ_1_ = 750 nm at ε = 0) of NR2-TP remains constant upon stretching, and a similar trend can also be found for NR2-LP. In addition, when the NRs’ size is increased from 200 to 250 nm of its center diameter, both NR3-TP and NR3-LP exhibit less sensitivity to the applied strains, where the three peaks seen at $\upvarepsilon =0$ show only slight fluctuations and weak merging trends. Nevertheless, one can observe a shift in the weights of the intensity for the main LSPR peaks of NR3 under both polarizations.

**Fig. 3:**
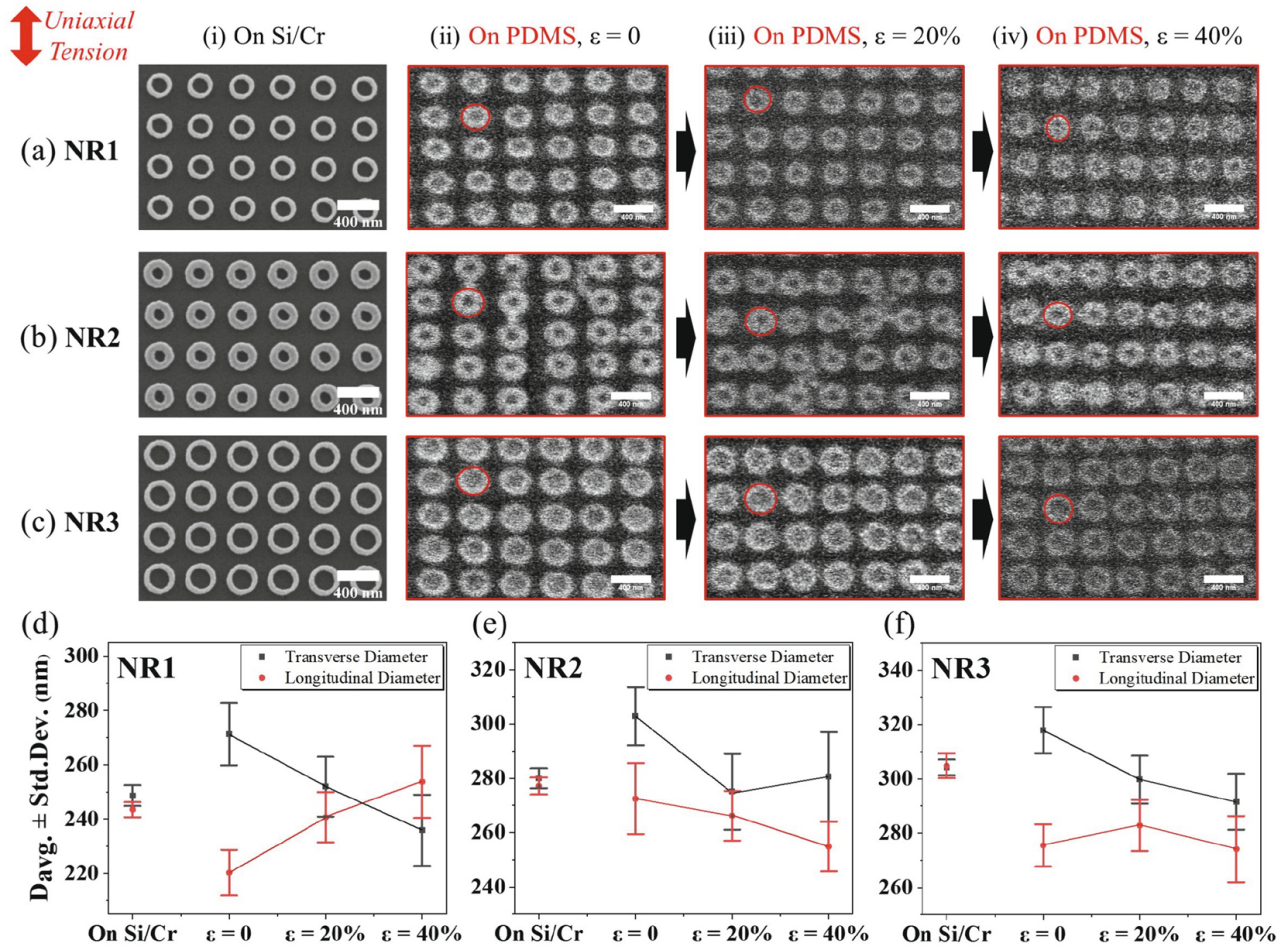
Set of SEM images of the three NR arrays studied in this work. Column (i) refers to the structures on Si/Cr before the transfer, while (ii-iv) show the SEM images on PDMS. The scale bar for all images is 400 nm, and the red lines outline the shapes of single NRs within their grating. (d–f) show the statistical evaluation of all NRs shown in (a–c), where the diameter here refers to the distance between the NRs’ outer walls

The results above indicate that the two arrays of thin NRs with different diameters present a similar redshift for the grating response, and the smaller NR1 exhibit a strong redshift for the main LSPR peaks, while the array of thicker NRs shows a reduced redshift for the grating mode, but unchanged LSPR modes under strain. These results indicate that NRs with a suitable thickness embedded in PDMS can make the substrate’s bulk shrinkage overcome the rigidity of the Au structure (with a modulus difference of ~ 69.1 GPa for nanostructured Au and ~ 2.6 MPa for PDMS), [48, 49] and enable an in-situ shape-altering under strain. We further perform an in-situ SEM characterization here to confirm the NRs’ deformability. To reduce the charge accumulating effect on non-conductive PDMS during the electron imaging, we first sputtered ~ 50 nm Au on its surface and then characterized the NR developments under each strain as shown in Fig. 3a, b and c. These figures still drift considerably over long measurement times, and to enable fast scanning, comparatively noisy screenshots are used to outline their shapes. Consequently, Fig. 3d–f plot the quantitative evaluation of the longitudinal/transverse (parallel/perpendicular to the uniaxial tensile direction) diameters of the three NR types on Si/Cr before the transfer and on PDMS under different strains. In all three arrays, the dimensions seem to have changed before and after the transfer, and the NRs on PDMS appear to be more elliptical. Afterward, the NR diameters in the NR1 array show a steady, approximately linear increase along with the stretching direction, and a decrease in the transverse direction. The increasing thickness of NR2 reduces their deformability with no clear trend within the error bars. NR3 shows a decreasing trend in the transverse direction and mostly unchanged longitudinal diameter as the strain increases, leading to a slightly increasing ratio between the longitudinal and transverse diameter. The reduced deformation of NR3 could be due to the reduced “free space” (i.e., grating period minus diameter) compared with NR1. It should be noted that due to the PDMS hardening effect and sputtered Au conductive layer, the deformability of the NRs on PDMS is significantly limited. We further fabricated another NR/PDMS system with similar geometries to NR1, and performed SEM tests at ε = 0 and the final ε = 30% state. An obvious shape development is then observed, which confirms the in-situ shape-altering for the NR1 array with strain applied to PDMS (Additional file 1: Fig. S2).

**Fig. 4:**
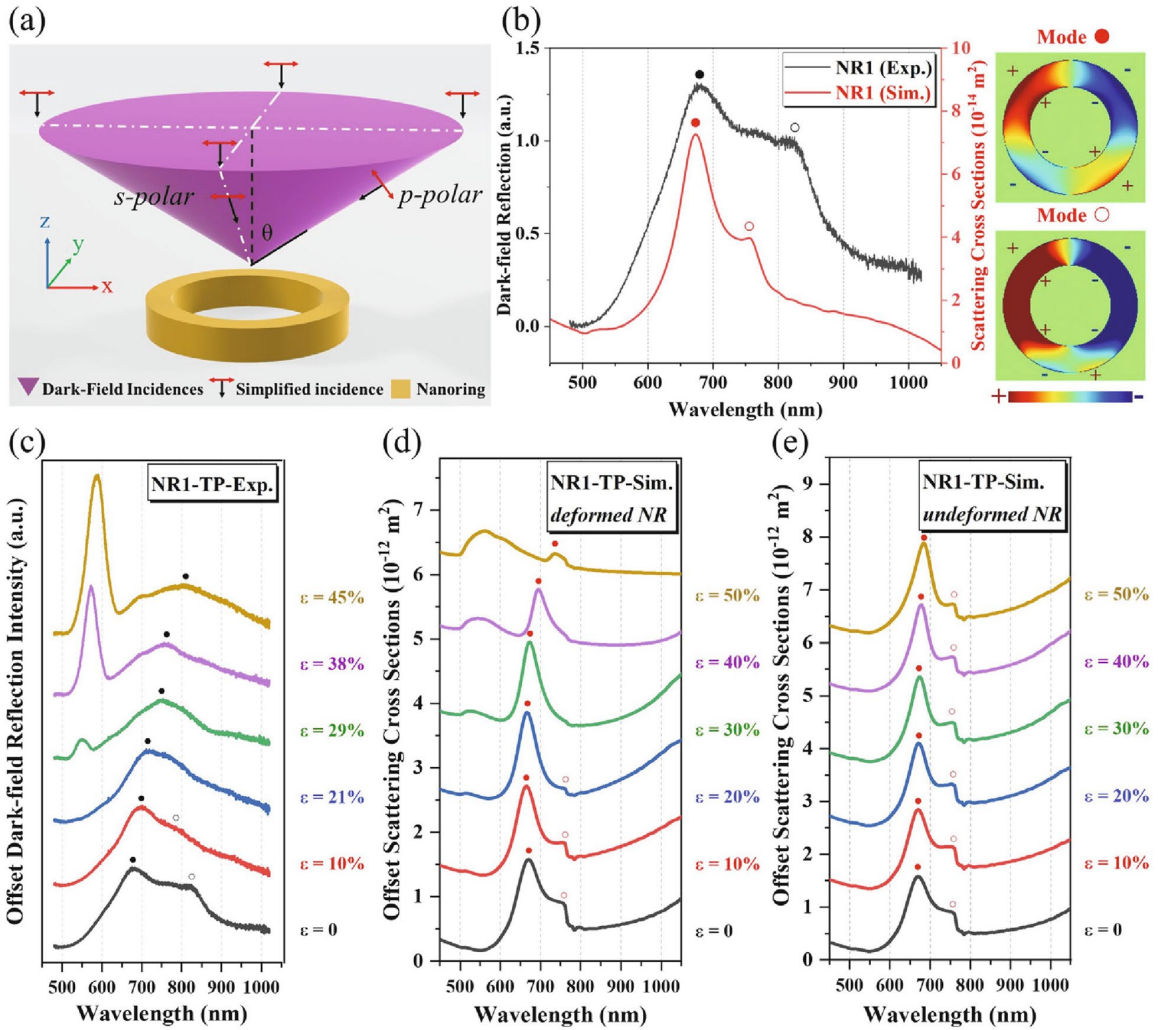
**a** Simplified schematic of dark-field incidence for simulations, where the cone-like dark-field incidence is formed through a ring-shaped condenser lens. **b** Comparison between spectra of the experiment and simulations for NR1-TP (left), and surface charge distributions for the two simulation LSPR modes (right). Comparison between NR1-TP spectra in experiments (**c**) and simulations of *deformed* (**d**) and *undeformed NRs* (**e**).

Next, we perform FDTD simulations to clarify the formation and redshift of the LSPR modes of the NR1 array. In literature, the standing-wave model is the common quantitative method to describe the LSPR peak of a linear antenna with a negligible thickness [25, 50, 51], i.e.,

$$\lambda {}_{LSPR} = \frac{2Ln}{m}$$

where L, m and n are the effective antenna length, bonding level (i.e. multipolar order, with m = 1 for dipolar modes, m = 2 for quadrupolar modes, etc.), and surrounding refractive index, respectively. In previous publications, researchers considered the NR as a “bending” nanorod, and suggested to replace L by an effective length L^*^ for NRs, which consists of either the center circle or the special ellipse connecting the NR’s outer and inner walls (Additional file 1: Fig. S3) [25, 51]. In case of the special ellipse approximation, the strain-induced shape-altering will lead to an increase of L^*^, resulting in a general redshift as a function of the NRs’ aspect ratio, independent of the polarization conditions [24, 25]. However, this equation particularly describes the radially symmetric bonding mode under normal incidence. The inherent oblique incidence from the dark-field illumination will excite additional LSPR modes for such a linear antenna (i.e., otherwise dark modes with even bonding level m become visible), especially for high curvature ones such as NRs [50]. Similar to literature [52] we approximate the polarized dark-field illumination as four partial characteristic oblique plane wave beams, as shown in Fig. 4a. The front and back beams are s-polarized (the E-fields are parallel to the object plane) in the x-direction, and the left and right ones exhibit an oblique p-polarization. Figure 4b plots a comparison between the spectra of NR1-TP from experiments and a single NR1 from simulations. The simulations indicate that two LSPR modes peaking at 675 and 759 nm correspond to radially symmetric and asymmetric charge density distributions with the same bonding level of m = 2, respectively. Furthermore, we perform two separate simulations concerning the deformability of the NR arrays under strain, i.e. deformed arrays with either deformed NRs or undeformed NRs, as shown in Fig. 4d and e, respectively. More simulation details can be found in Additional file 1: Figs. S4–S6. By assuming the NR is deformable under strain, Fig. 4d shows a disappearing trend for the second LSPR mode (marked with open circles) as strain develops (0 ≤ ε ≤ 30%), and a gradual redshift for the main LSPR mode (solid circles) under high strain values (ε ≥ 30%). These behaviors qualitatively reproduced the experimental results of NR1-TP as shown in Fig. 4c. By contrast, the two LSPR modes in Fig. 4e remain generally constant over strains, which is more in line with the spectra of the thicker NR arrays, such as NR2-TP in Fig. 2a. These results illustrate that the NR1 array shows a general redshift for the radially symmetric bonding mode, while the asymmetric mode tends to disappear or merge with the main LSPR mode as its shape develops under strain.

## Conclusions

To conclude, we move beyond traditional lithography on a rigid substrate and fabricate a series of Au NR arrays on the flexible substrate PDMS via state-of-the-art EBL and a pattern transfer technique. We demonstrate the shape-altering flexible plasmonics of in-situ deformable NRs induced by the stretching of the PDMS. The shape development from initially regular NRs to elliptical ones for the array of the thin NR1 has been shown to act as an effective strain sensor with a stronger spectral redshift for the main plasmonic mode, and thus higher sensitivity, than previous gap-altering flexible devices. Further in-situ SEM tests confirm that the shape-altering significantly depends on the NRs' thickness. The array of the thicker NR2 presents no obvious shape development under strain due to the increase in ring thickness and thus shows only a less strongly red-shifting lattice resonance and a constant plasmonic mode. By comparing the experimental spectrum with numerical simulations, we explain that the spectral shift of the thin NR1 array results from its radially symmetric bonding mode, whereas the asymmetric mode tends to disappear as the shape develops under strain. Such interesting spectral tunability makes NRs a promising platform for strain-/bio-sensing applications. In future work, the shifting modes could be further distinguished e.g. by removal of the grating mode and spectral tuning within the visible region.

## Supplementary Information

**Additional file 1: Fig. S1.** Dark-field reflection spectra for three disc arrays with increasing sizes. **Fig. S2.** Shape-altering of a second nanoring array. **Fig. S3.** Effective length models for the nanorod and nanoring. **Figs. S4-S6.** Supplementary simulations.

## Data Availability

The datasets used and/or analyzed during the current study are available from the corresponding author on reasonable request.

## Abbreviations

NRs: Nanorings
LSPRs: Localized surface plasmon resonances
NPs: Nanoparticles
PH: Plasmon hybridization
EBL: Electron beam lithography
ENRs: Elliptical nanorings
PDMS: Polydimethylsiloxane
SEM: Scanning electron microscope
FDTD: Finite-difference time-domain
Au: Gold
Cr: Chromium
Si: Silicon
PMMA: Poly-methyl methacrylate
MIBK: Methyl isobutyl ketone
MPTS: (3-Mercaptopropyl) triethoxysilane
SD: Standard deviation
TP: Transverse polarization
LP: Longitudinal polarization
