# Supplementary material for: Shape-altering flexible plasmonics of in-situ deformable nanorings
Source: Nano Converg. 2023 Mar 30;10:15. doi: 10.1186/s40580-023-00358-6 (PMC10063774; doi:10.1186/s40580-023-00358-6)
Supplement: Supplementary file 1 — Additional file 1: Fig. S1. Dark-field reflection spectra for three disc arrays with increasing sizes. Fig. S2. Shape-altering of a second nanoring array. Fig. S3. Effective length models for the nanorod and nanoring. Figs. S4-S6. Supplementary simulations. [file 40580_2023_358_MOESM1_ESM.pdf]

Supplementary Information:

Shape-altering Flexible Plasmonics of In-situ Deformable Nanorings

Wei Tao,[1,2,*] Florian Laible,[1] Abdelhamid Hmima,[2] Thomas Maurer[2,*] and Monika Fleischer[1,*]

[1] Institute for Applied Physics and Center LISA+, Eberhard Karls University Tübingen, 72076 Tübingen, Germany

[2] Laboratory Light, Nanomaterials and Nanotechnologies—L2n, University of Technology of Troyes and CNRS EMR 7004, 12 rue Marie Curie, CS 42060, CEDEX, 10004 Troyes, France

* indicates the corresponding author

*Corresponding authors: Wei Tao, Thomas Maurer, Monika Fleischer

E-mail address: *wei.tao@utt.fr; thomas.maurer@utt.fr; monika.fleischer@uni-tuebingen.de*

Fig. S1. Dark-field reflection spectra for three disc arrays with increasing sizes.

To distinguish the Bragg reflection mode and plasmonic mode, we fabricate a set of disc arrays on a rigid substrate of indium tin oxide/glass via a similar lithography technique as introduced in the main text. Note that these arrays are of a constant lattice period of 500 nm, a height of 50 nm, and increasing diameters of (173.0 ± 3.3) nm, (188.1 ± 3.3) nm, and (202.5 ± 6.6) nm, as shown in Fig. S1 (a), (b) and (c), respectively. The corresponding dark-field reflection spectra for the three arrays are characterized under unpolarized light via the same microscope setup. On the one hand, the right peak in Fig. S1(d) exhibits a clear redshift from 820 to 858 nm as the disc size increases, indicating it as the dipolar plasmonic disc mode. [1] On the other hand, since the period is increased from 400 nm in the main text to 500 nm here, a broader mode is observed peaking at ~575 nm, which does not shift as the disc size increases. This typical spectral behavior is in line with a Bragg grating mode, where the position depends only on the refractive index of the medium and the lattice period, and an increasing geometric size should only influence the spectral line width. [2,3] Furthermore, based on the redshift of the mode marked by a triangle in Fig. 2 in the main text, one can expect a continuous tuning of this peak from the ultra-violet to ~600 nm for increasing lattice periods. Since Au structures with geometric dimensions over 200 nm can hardly achieve a dipolar plasmonic resonance at such short wavelengths it is thus confirmed that the peaks marked by triangles in Fig. 2 correspond to the Bragg reflection mode. [4,5]

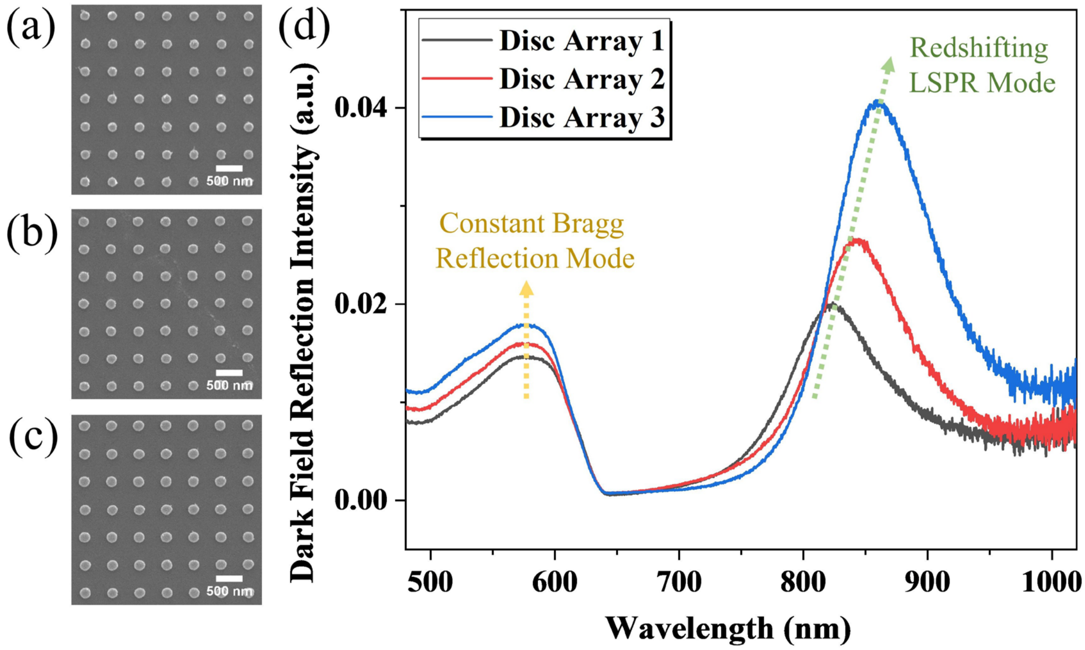

Fig. S1 Set of disc arrays on an ITO/glass substrate, with respective diameters of (173.0 ± 3.3) nm (a); (188.1 ± 3.3) nm (b); and (202.5 ± 6.6) nm (c). Dark-field reflection spectra of the three arrays (d).

Fig. S2. Shape-altering of a second nanoring (NR) array.

As a supplement to the deformable NR1 array in the main text, we also fabricated another NR array (with similar dimensions) on PDMS via the same electron beam lithography (EBL) and transfer techniques. Such a sample is directly used to perform the scanning electron microscopy (SEM) test after sputtering ~50 nm Au on top of the structured PDMS. Fig. S2 (a) shows the NR array on PDMS at ε = 0 (without strain), where (b) and (c) show the same NR array at ε = 30% in different zones. A clear shape-altering from the initial circular to the final elliptical NRs due to the applied strain can be seen. The white frame boxes outline a series of NRs within the array, indicating an increasing aspect ratio (i.e., ratio of the NR's outer diameter in the direction of strain to that perpendicular to the strain) from ~1.02 to 1.15 at ε = 0 and 30%, respectively. Besides, the uniaxial splitting of the sputtered Au layer in Figs. S2(b) and (c) (see horizontal cracks) also illustrates that the shape development is partially restricted.

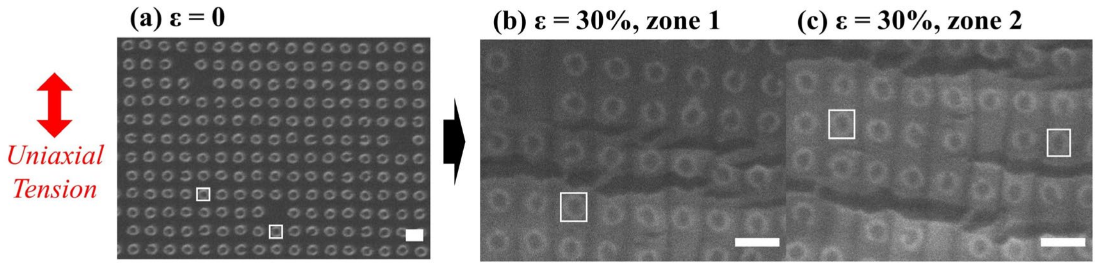

Fig. S2 Top-view images of the supplementary NR array on PDMS at a strain of ε = 0 (a) and 30% (b and c). The scale bar for the three images is fixed at 400 nm.

Fig. S3. Effective length models for the nanorod and NR.

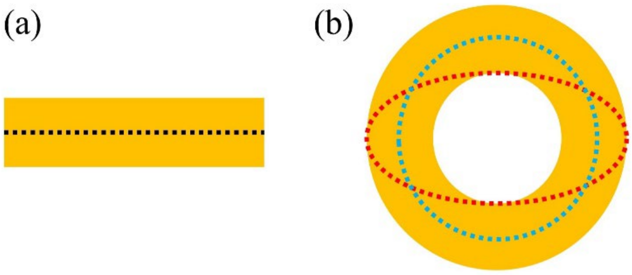

Fig. S3 Schematic of effective length models for the nanorod (a) and NR (b), where red and blue dot-lines denote the path of the charges as a center circle or a special ellipse connecting the NR's outer and inner walls, following references by Apter et al. [6] and Cai et al. [7]

Fig. S4-S6. Supplementary simulations.

Here, we present two more strategies to simulate the dark-field spectra of a single NR1 structure, i.e. single-beam s-polarized incidence (θ = 56.75°), and normal incidence (θ = 0°), as shown in Fig. S4. The s-polarized beams are schematically shown in Fig. 4 (a) in the main text. In the near-infrared region, the NRs under both normal and single s-polarized incidence show a peak at the wavelength of ~1050 nm referring to a typical dipolar bonding ring mode. Focusing on the detection range in the experiments (480-1020 nm), the NR1 under normal incidence shows no LSPR peaks, while the simulation spectra show two LSPR modes under single-beam s-polarization similar to the main text.

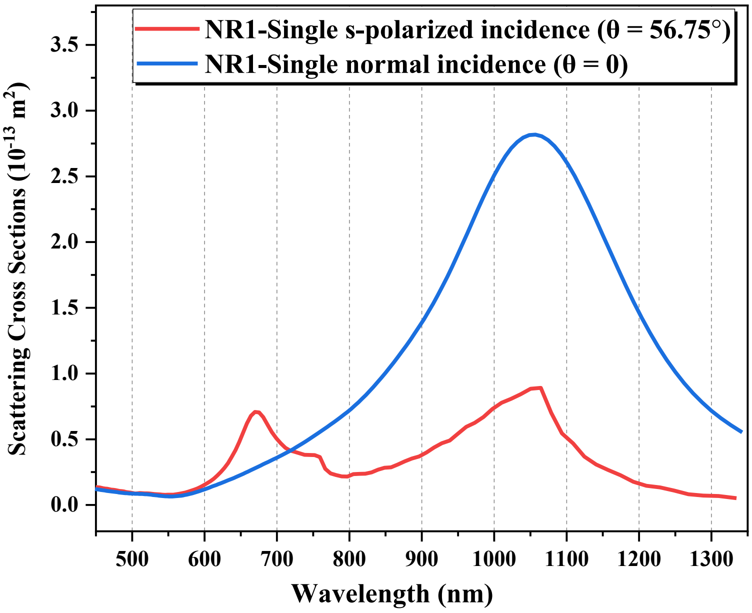

Fig. S4 Comparison of simulation spectra for a single NR1 under s-polarized tilted incidence and under normal incidence illumination.

In order to simulate the spectral behavior of the NR1 array under strain, we first discuss the strain-induced variations in the lattice period on PDMS. In line with literature, we consider uniform strain-induced periodicity variations, where the NRs' longitudinal and transverse center-to-center spacing (period) under strain (Px^{ε} and Py^{ε}) follows an elastic model: [8,9]

$$Px^{\varepsilon} = Px(1 + \varepsilon)$$

$$Py^{\varepsilon} = Py(1 - \nu\varepsilon)$$

where ν denotes a constant Poisson ratio of PDMS of 0.5. The continuous tuning of the array's period can be confirmed by either the gradual redshift of the Bragg mode or the direct observation during the optical measurements.

On the other hand, in the main text and Fig. S2, we performed SEM tests to illustrate the shape-altering of NR1 under strain. However, recording the exact shape development at each strain is challenging since the NRs' deformability is significantly reduced under SEM imaging by either the hardening effect of PDMS under the electron beam or a sputtered conductive layer. Therefore, we perform two separate simulations regarding the deformability of the NR arrays under strain, i.e. deformed arrays with either *deformed NRs* or *undeformed NRs*. A finite 5 x 5 array is defined to simulate the experimental lattice, where the periodicities for the two cases develop elastically under the strain as shown in Fig. S5.

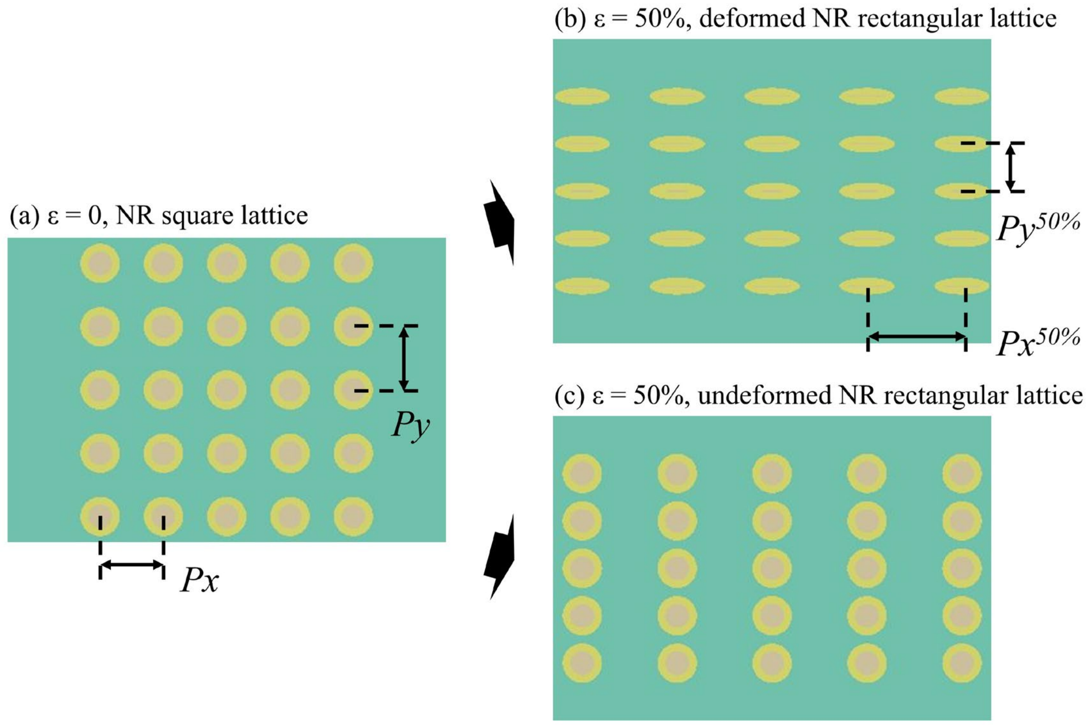

Fig. S5 The elastic grating development of a finite 5 x 5 NR array in simulations, where figure (b) assumes the NRs are deformed within the array, and (c) suggests undeformed NRs under strain. The images are taken from the software layout of *Lumerical FDTD solutions*.

Furthermore, we make a few assumptions for the simulation studies: (1) a single s-polarized beam is introduced to simulate the transverse-polarized experimental dark-field incidence; (2) the thickness of the NRs remains unchanged under strain; (3) for the case of *deformed NRs*, the center diameter (long axis) parallel to the stretching (Dx^{ε}) deforms elastically; and (4) the center perimeter of the NRs (P) remains unchanged with strain, which tailors the center diameter (short axis) perpendicular to the strain (Dy^{ε}). The shape-altering is quantitatively approximated by the

following equations and qualitatively depicted in Fig. S6. (The perimeter of the ellipse is approximated by the Euler equation of $P \approx \pi*(2(a^2+b^2))^{0.5}$. [10])

$$Dx^{\varepsilon} = Dx(1+\varepsilon)$$

$$Dy^{\varepsilon} \approx \sqrt{\frac{P^2}{2\pi^2} - \left(Dx^{\varepsilon}\right)^2}$$

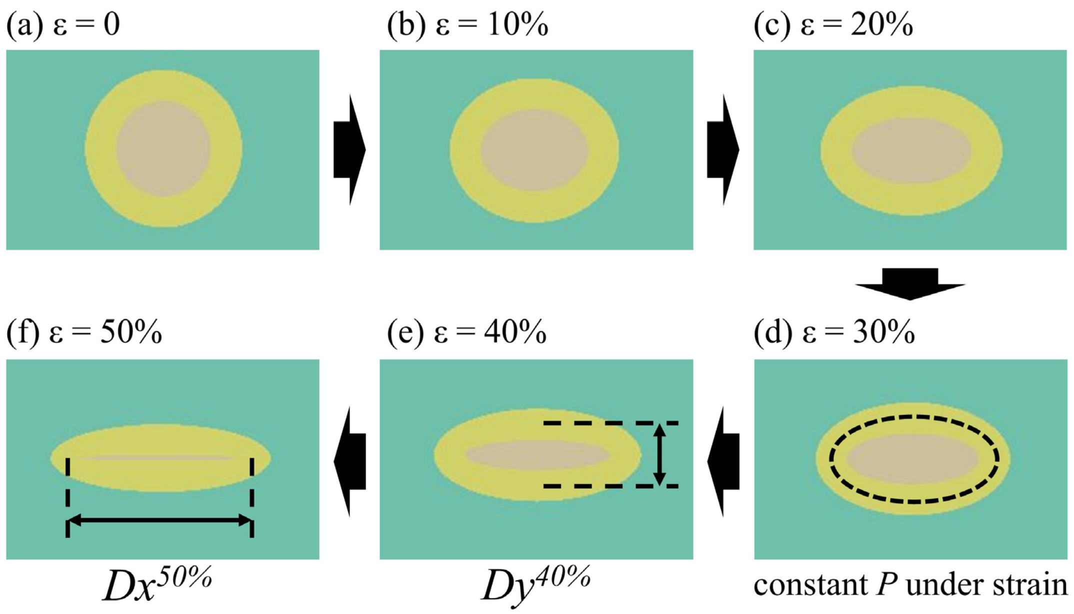

Fig. S6 The shape-altering of a single NR within its array in simulations. The images are taken from the software layout of *Lumerical FDTD solutions*.

Finally, Fig. 4 (c), (d) and (e) in the main text shows the comparison of experimental spectra of the NR1 array with the two simulations under transverse polarization. Despite the narrower linewidth, the simulation results of *deformed NRs* qualitatively show the characteristics of the experimental curves, e.g., (1) the LSPR modes show lower intensities under higher strains; (2) the main LSPR mode (marked with solid circles) shifts little or remains constant at the beginning, but presents stronger redshifts at higher ε; and (3) the second mode (open circles) tends to disappear as strain increases. However, the simulation results of *undeformed NRs* show only a slight redshift for the main LSPR mode from ~670 to 683 nm at ε = 0 and 30% respectively, and a constant second mode over the different strains, which rather corresponds to the behavior of the thicker NR2. These results indicate that the substantial redshift and merging trend for the NR1 arrays under strain stems from the NRs' in-situ shape-altering instead of variations of lattice periods, and further confirm the strain-induced shape-altering for the NR1 array, while the behavior of NR2 is more in line with undeformed NRs.

References

1. L. Jiang *et al.*, ACS nano **9**, 10039 (2015).

2. V. Gupta, P. T. Probst, F. R. Goßler, A. M. Steiner, J. Schubert, Y. Brasse, T. A. König, and A. Fery, ACS applied materials & interfaces **11**, 28189 (2019).

3. A. D. Utyushev, V. I. Zakomirnyi, A. E. Ershov, V. S. Gerasimov, S. V. Karpov, and I. L. Rasskazov, Photonics **7**, 24 (2020).

4. S. Link and M. A. El-Sayed, The Journal of Physical Chemistry B **103**, 4212 (1999).

5. N. Cathcart, J. I. Chen, and V. Kitaev, Langmuir **34**, 612 (2018).

6. B. Apter, O. Guilatt, and U. Efron, Applied Optics **50**, 5457 (2011).

7. Y. Cai, Y. Li, P. Nordlander, and P. S. Cremer, Nano letters **12**, 4881 (2012).

8. A. Yang, A. J. Hryn, M. R. Bourgeois, W.-K. Lee, J. Hu, G. C. Schatz, and T. W. Odom, Proceedings of the National Academy of Sciences **113**, 14201 (2016).

9. Y. Brasse, V. Gupta, H. T. Schollbach, M. Karg, T. A. König, and A. Fery, Advanced Materials Interfaces **7**, 1901678 (2020).

10. R. W. Barnard, K. Pearce, and L. Schovanec, Journal of mathematical analysis and applications **260**, 295 (2001).
